# Vaccination against a hit-and-run viral cancer

**DOI:** 10.1099/vir.0.023507-0

**Published:** 2010-09

**Authors:** Philip G. Stevenson, Janet S. May, Viv Connor, Stacey Efstathiou

**Affiliations:** Division of Virology, Department of Pathology, University of Cambridge, UK

## Abstract

Cancers with viral aetiologies can potentially be prevented by antiviral vaccines. Therefore, it is important to understand how viral infections and cancers might be linked. Some cancers frequently carry gammaherpesvirus genomes. However, they generally express the same viral genes as non-transformed cells, and differ mainly in also carrying oncogenic host mutations. Infection, therefore, seems to play a triggering or accessory role in disease. The hit-and-run hypothesis proposes that cumulative host mutations can allow viral genomes to be lost entirely, such that cancers remaining virus-positive represent only a fraction of those to which infection contributes. This would have considerable implications for disease control. However, the hit-and-run hypothesis has so far lacked experimental support. Here, we tested it by using Cre–*lox* recombination to trigger transforming mutations in virus-infected cells. Thus, ‘floxed’ oncogene mice were infected with Cre recombinase-positive murid herpesvirus-4 (MuHV-4). The emerging cancers showed the expected genetic changes but, by the time of presentation, almost all lacked viral genomes. Vaccination with a non-persistent MuHV-4 mutant nonetheless conferred complete protection. Equivalent human gammaherpesvirus vaccines could therefore potentially prevent not only viral genome-positive cancers, but possibly also some cancers less suspected of a viral origin because of viral genome loss.

## INTRODUCTION

The identification of viral aetiologies for hepatic (Blumberg, 1997) and cervical (Frazer, 2004) cancers has made antiviral vaccination a relatively simple and effective means of disease prevention. Human gammaherpesviruses – Epstein–Barr virus (EBV) and Kaposi's sarcoma-associated herpesvirus (KSHV) – are also oncogenic, but the lack of single, unifying features of the associated cancers has made it unclear how directly infection and disease are linked and so what vaccination might achieve. The robust persistence of herpesviruses in immunocompetent hosts also makes vaccination a considerable challenge.

EBV transforms B cells *in vitro* and, in immunocompromised patients, the viral genes responsible for transformation can cause disease (Carbone *et al.*, 2009). However, EBV-infected cancers in immunocompetent hosts tend to express the same viral genes as non-transformed cells. They differ in also carrying oncogenic host mutations; indeed, Burkitt's lymphoma is associated more strongly with c-myc translocation than with EBV infection (Thorley-Lawson & Allday, 2008). Thus, viral genes seem mostly to have triggering or accessory roles in disease, with host oncogenes being the main drivers. The hit-and-run hypothesis proposes that viral genomes initiating disease can be lost entirely to obscure a cancer's viral origin (Ambinder, 2000). Early on, viral genes are likely to be essential for cancer-cell survival (Hammerschmidt & Sugden, 2004). However, cancers accumulate vast numbers of host mutations (Pleasance *et al.*, 2010), some of which will inevitably promote more autonomous growth. Thus, it seems inevitable that a cancer will, with time, evolve increasing independence from viral gene functions that could allow viral genome loss.

The main problem with the hit-and-run hypothesis has been a lack of experimental support. Analyses of gammaherpesvirus-induced cancers have focused on African Burkitt's lymphoma, nasopharyngeal carcinoma and Kaposi's sarcoma, because their high frequencies of viral genome retention make plausible a causal link between infection and disease. However, focusing on virus-positive cancers tells us little about genome loss, as here most presenting cancers would be virus-negative. Instead, it is necessary to track prospectively the fate of viral genomes in transformed cells. *In vitro*, B-cell cancers tend to maintain gammaherpesvirus genomes, whereas Kaposi's sarcoma and nasopharyngeal carcinoma tend to lose them (Ganem, 2006; Dittmer *et al.*, 2008). *In vivo*, murid herpesvirus-4 (MuHV-4) infection increases the incidence of virus-negative cancers (Sunil-Chandra *et al.*, 1994; Tarakanova *et al.*, 2005). However, the difficulty of analysing spontaneous cancers, where the molecular changes driving transformation are almost always unknown, makes firm functional conclusions hard to draw. To ensure that the host factors contributing to cancer remained known, we used Cre–*lox* recombination in a well-established conditional mouse cancer model (reviewed by DuPage *et al.*, 2009) to transform virus-infected cells, and then analysed the emerging cancers for viral genome retention.

## RESULTS

### Generation of Cre^+^ MuHV-4

**Fig. 1.**
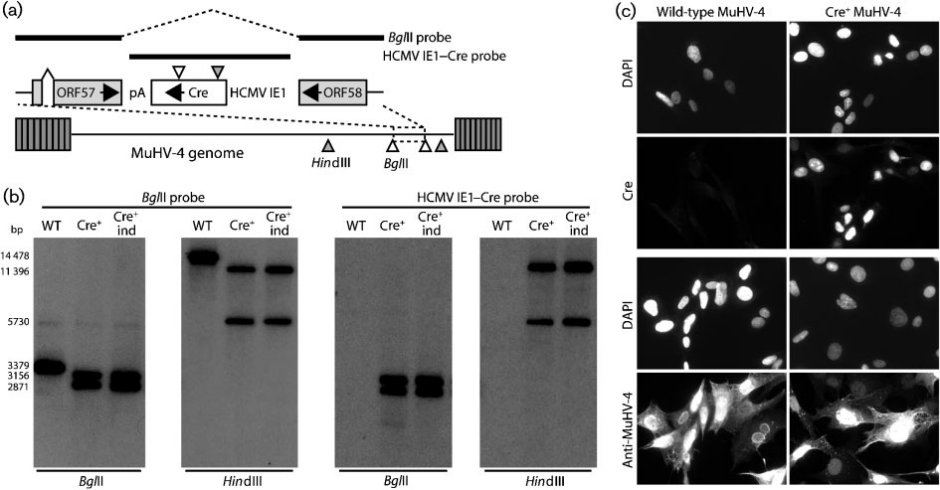
Characterization of Cre^+^ MuHV-4. (a) An HCMV IE1 promoter-driven Cre expression cassette was inserted between MuHV-4 ORFs 57 and 58. Relevant restriction sites are shown. (b) Viral DNA was digested with *Hin*dIII or *Bgl*II and probed with either a genomic *Bgl*II clone or the HCMV IE1–Cre construct, as shown in (a). WT, Wild-type; Cre^+^, recombinant; Cre^+^ind, independently derived recombinant. (c) BHK-21 cells were infected with wild-type or Cre^+^ MuHV-4 (1 p.f.u. per cell, 16 h), then fixed, permeabilized and stained for Cre recombinase or for MuHV-4 antigens using polyclonal rabbit sera. Nuclei were counterstained with DAPI.

We inserted a human cytomegalovirus (HCMV) IE1 promoter-driven Cre expression cassette between the 3′ ends of MuHV-4 ORFs 57 and 58 (Fig. 1a, b). We used an HCMV IE1 promoter because this can be active in latently infected cells (Rosa *et al.*, 2007; Smith *et al.*, 2007). Thus, Cre could be expressed without MuHV-4 lytic genes killing the infected cells. Two functionally indistiguishable mutants were obtained. Both showed Cre expression by excising spontaneously their *loxP*-flanked bacterial artificial chromosome (BAC) cassettes, and immunofluorescence showed Cre expression in infected-cell nuclei (Fig. 1c). (The Cre coding sequence used incorporates an N-terminal nuclear-localization signal.)

### *In vivo* *loxP* recombination by Cre^+^ MuHV-4

**Fig. 2.**
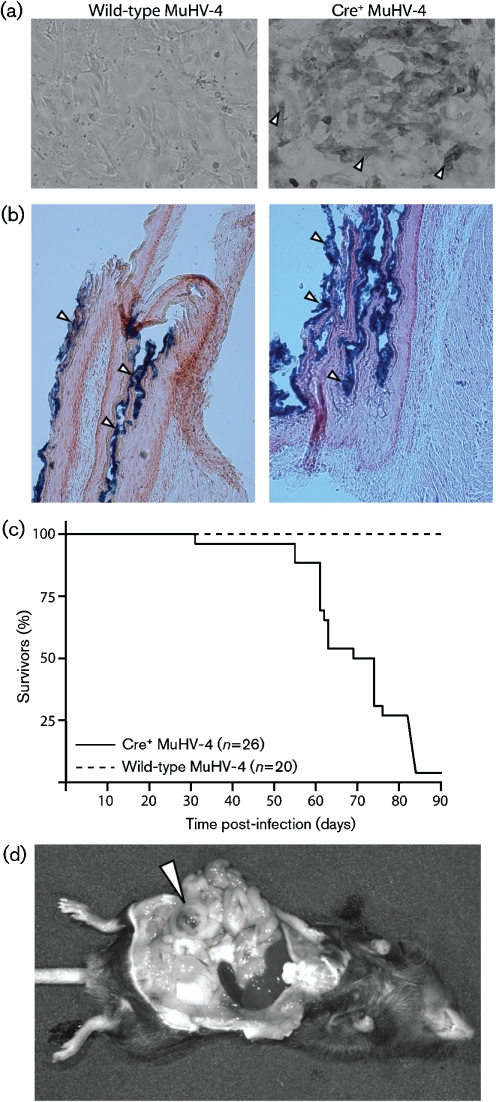
Cre recombinase-triggered cancers in MuHV-4-infected mice. (a) Embryonic fibroblasts from ROSA26-*lacZ*^flox/flox^ mice were infected (0.3 p.f.u. per cell, 16 h) with either wild-type or Cre^+^ MuHV-4, then fixed and incubated with X-Gal to reveal *β*-galactosidase expression, indicating Cre-mediated recombination. Arrowheads show examples of positive staining. (b) ROSA26-*lacZ*^flox/flox^ mice were infected i.p. with Cre^+^ MuHV-4. Three days later, diaphragms were stained post-mortem for *β*-galactosidase expression with X-Gal. Representative images from two mice are shown. (c) p53^flox/flox^K-ras^LSL-G12D/+^ mice were infected i.p. with wild-type or Cre^+^ MuHV-4. All of the former mice remained healthy; all but one of those infected with Cre^+^ MuHV-4 developed cancers within 3 months. Equivalent results were obtained in three further experiments. (d) A typical i.p. cancer.

We tested whether viral Cre expression could recombine *loxP* sites in the host genome by infecting mouse embryonic fibroblasts derived from ROSA26-*lacZ*^flox/flox^ reporter mice (Fig. 2a). *β*-Galactosidase assays were strongly positive, indicating *loxP* recombination. Such recombination was also achieved by infecting ROSA26-*lacZ*^flox/flox^ mice intraperitoneally (i.p.) with Cre^+^ MuHV-4 (Fig. 2b): widespread *β*-galactosidase expression was evident on the diaphragm, a site commonly infected by i.p. MuHV-4 (Milho *et al.*, 2009).

We then infected p53^flox/flox^K-ras^LSL-G12D/+^ mice i.p. with Cre^+^ MuHV-4 (Fig. 2c, d). More than 90 % of infected mice developed cancers within 3 months, compared with 0 % of uninfected or wild-type MuHV-infected controls. Cancers occurred most frequently on the diaphragm. Disease was rare within 30 days, and most cancers were single lesions. In contrast, virus replication was widespread: 3 days after inoculation, spleens yielded (2.1±1.2)×10^4^ and peritoneal washes (1.7±1.2)×10^3^ infectious centres per mouse (mean±sd titres, *n*=6, with lytic titres <1 % of infectious centre titres); even 2 months later, spleens yielded (2.2±1.5)×10^2^ infectious centres per mouse (*n*=6). Therefore, cancer growth was much more restricted than viral latency and functional Cre expression.

### Analysis of virus-triggered cancers

**Fig. 3.**
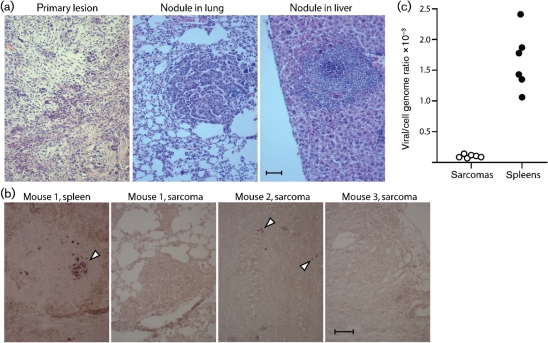
MuHV-4-triggered sarcomas. (a) Representative haematoxylin/eosin-stained sections from p53^flox/flox^K-ras^LSL-G12D/+^ mice infected with Cre^+^ MuHV-4. Bar, 100 μm. (b) Cancer or spleen sections of Cre^+^ MuHV-4-infected p53^flox/flox^K-ras^LSL-G12D/+^ mice were probed for MuHV-4 tRNAs 1–4. Representative images are shown. Arrowheads show positive cells. Bar, 100 μm. (c) DNA samples from paired cancers and spleens were analysed for viral genome copy number by quantitative PCR. Each viral copy number is expressed relative to the cellular DNA copy number in the same sample.

All of the cancers analysed (*n*>12) were histological sarcomas (Fig. 3a). *In situ* hybridization (Fig. 3b) showed surprisingly little expression of the MuHV-4 tRNAs normally abundant in lytic and latent infections (Bowden *et al.*, 1997). At most, a few positive cells were scattered around the main cancer mass. Real-time PCR (Fig. 3c) established that sarcomas contained lower copy numbers of viral genomes than latently infected spleens of the same mice.

**Fig. 4.**
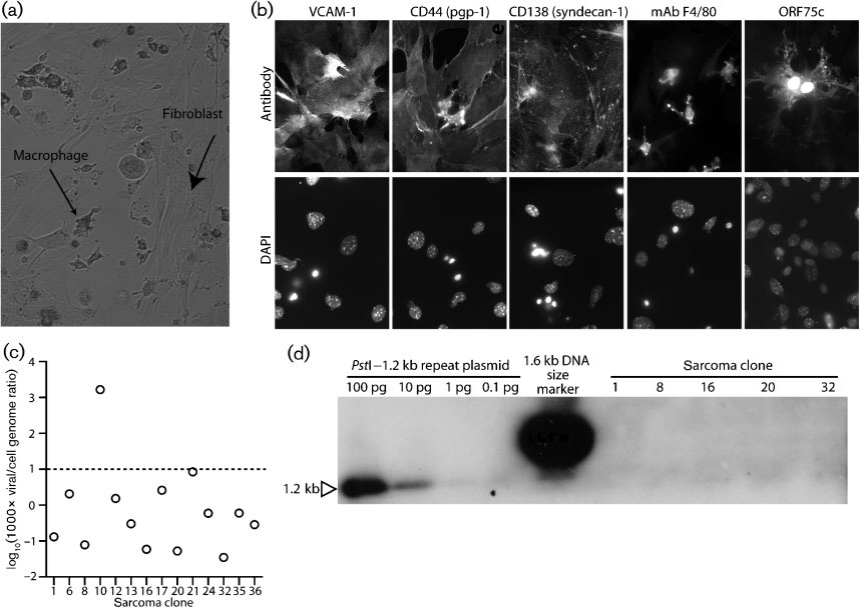
Analysis of explanted cancer cells from Cre^+^ MuHV-4-infected p53^flox/flox^K-ras^LSL-G12D/+^ mice. (a) A typical phase-contrast image of a primary cancer culture 1 day post-explant. (b) Immunostaining of a primary cancer culture at 3 days post-explant shows typical VCAM-1^+^CD44^+^CD138^+^ fibroblasts, and some F4/80^+^ macrophages. Occasional fibroblasts (<1 %) were viral antigen-positive, shown here by staining for the ORF75c tegument protein. (c) Cloned cancer cells were analysed for viral genomes by quantitative PCR. Viral DNA copy numbers are expressed relative to cellular DNA copy numbers. Only clone 10 yielded infectious virus; below the dashed line (<1 viral genome per 100 cell genomes), clones were considered virus-negative. (d) A subset of the clones in (c) was further analysed by probing *Pst*I-digested DNA (1 mg per lane=500 000 cells) for the MuHV-4 1.2 kb terminal repeat (approx. 30 copies per genome) by Southern blotting. One picogram of plasmid DNA=200 000 copies, so no detectable viral genomes implies <1 copy per 75 cells.

**Fig. 5.**
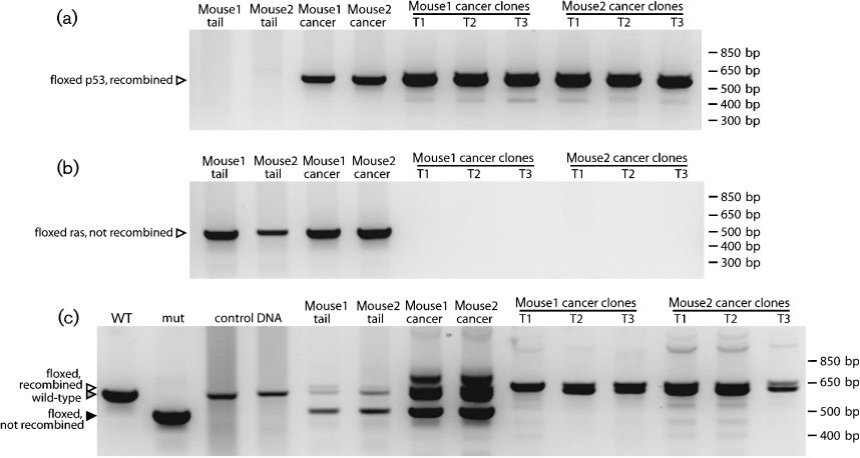
PCR detection of Cre-mediated recombination in samples from Cre^+^ MuHV-4-infected p53^flox/flox^K-ras^LSL-G12D/+^ mice. (a) PCR analysis of the p53 locus of two p53^flox/flox^K-ras^LSL-G12D/+^ mice, their primary cancers and fibroblast clones derived from them. The primers amplify the floxed p53 locus only after recombination (612 bp). Identical data were obtained for a further 10 mice. Negative images of ethidium bromide-stained PCR products are shown. (b) PCR analysis of the floxed G12D k-ras cassette of the same samples. The primers amplify the cassette (550 bp) before but not after recombination. (c) Multiplex PCR analysis of the ras locus of the same samples plus additional controls. The primers amplify from the wild-type k-ras locus a 622 bp band, and from the floxed G12D k-ras cassette a 500 bp band before recombination and a 650 bp band after recombination. The clones lack the 500 bp band of the parental cancers because they contain no cells with unrecombined G12D k-ras. WT, p53^flox/flox^G12D k-ras^−/−^ littermate; mut, purified 500 bp band; control DNA, non-transgenic mice.

Fresh sarcoma explants included lymphocytes, macrophages and fibroblasts (Fig. 4a, b), but only fibroblasts grew out. Thirteen of 20 explants yielded infectious virus. Viral spread soon overwhelmed these positive cultures, consistent with fibroblasts being highly permissive for MuHV-4 lytic replication. The others remained virus-negative. At 2 days post-explant, titres were low in all cultures (<1 p.f.u. per 10^4^ cells), and <5 % of fibroblasts cloned at this time (39 of 744 clones from eight mice) yielded infectious virus. Clones lacking infectious virus also lacked viral genomes by PCR (Fig. 4c) and Southern blotting (Fig. 4d). Nevertheless, all sarcomas showed the expected patterns of Cre-induced p53 disruption and k-ras(G12D) expression (Fig. 5). Therefore, the vast majority of cancer cells showed genetic changes consistent with previous virus infection but, by the time of presentation, were not virus-infected.

A trivial explanation for the lack of viral genomes in transformed cells would be that Cre uptake from infected-cell debris was sufficient for transformation. However, infecting p53^flox/flox^K-ras^LSL-G12D/+^ mice (*n*=24) with herpes simplex virus (HSV) expressing Cre from an HCMV IE1 promoter caused no disease. Also, Cre^+^ HSV similarly shows no spread of Cre signal *in vivo* (Proença *et al.*, 2008), and Cre^+^ MuHV-4 plaque assays on ROSA26-*lacZ*^flox/flox^ fibroblasts showed no obvious spread of *β*-galactosidase expression to uninfected cells.

Even when virus was recovered from cancer cells, it might have come from infiltrating, non-transformed cells rather than being that responsible for the original oncogenic hit. We examined this possibility by infecting mice with a mix of Cre^+^ and Cre^−^ MuHV-4 and typing the virus recovered from sarcomas for Cre expression. Cre^+^ MuHV-4 showed approximately 30-fold lower latent titres than Cre^−^ virus, so we used an input Cre^+^/Cre^−^ mixture of 30 : 1. Only one of 18 virus-positive sarcoma explants was Cre^+^ by immunofluorescence. PCR and DNA sequencing of the ORF57/58 junction showed that the Cre^−^ viruses were wild-type. This did not cause sarcomas (Fig. 2), so even when virus infection was observed in sarcoma explants, it appeared rarely to be that responsible for transformation.

### Vaccination against virus-triggered cancers

**Fig. 6.**
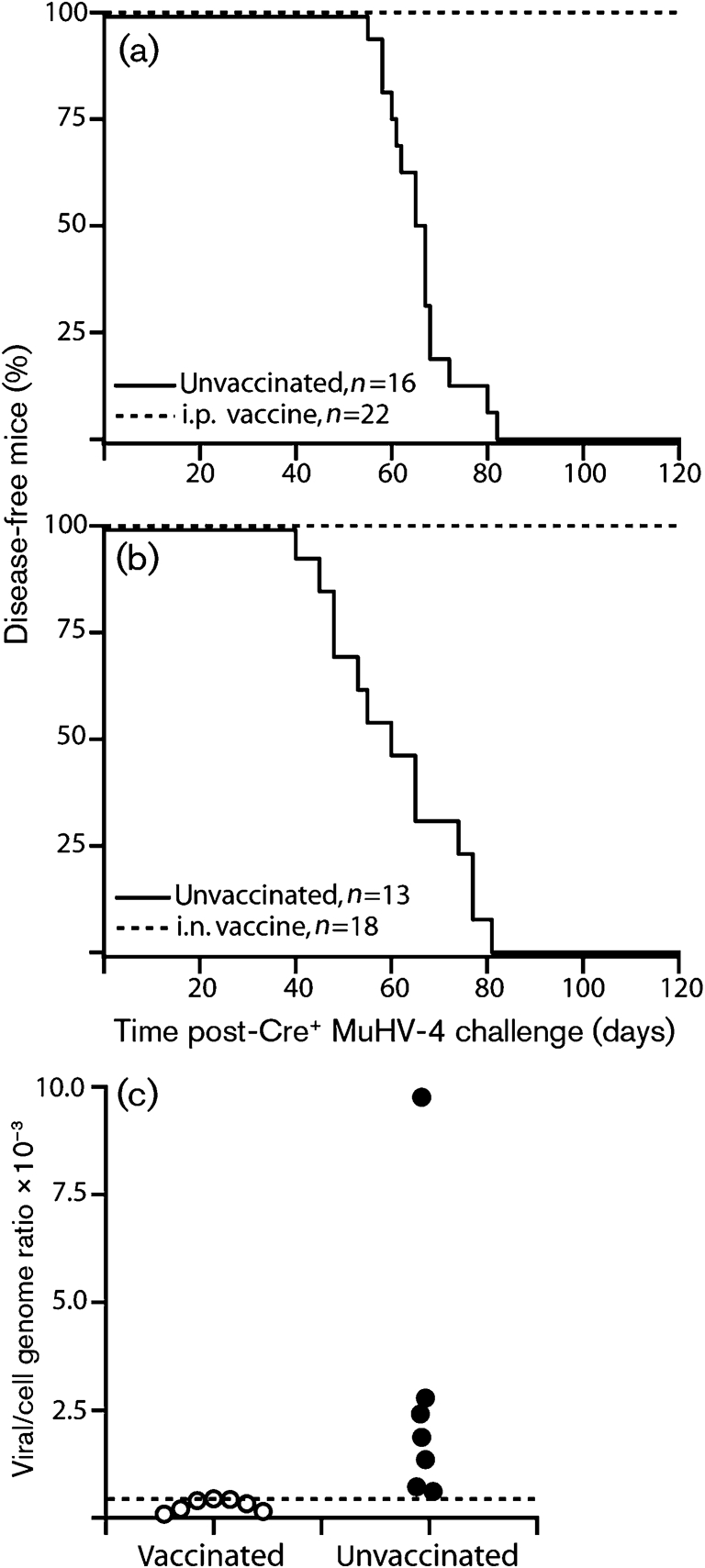
Vaccination against MuHV-4-triggered sarcomas. (a) p53^flox/flox^K-ras^LSL-G12D/+^ mice were not vaccinated or vaccinated i.p. with ORF73^−^Cre^−^ MuHV-4, then 2 months later challenged i.p. with Cre^+^ MuHV-4 and followed for cancer incidence. At 4 months, the vaccinated mice showed no disease. The data are from one of two equivalent experiments. (b) p53^flox/flox^K-ras^LSL-G12D/+^ mice were not vaccinated or vaccinated i.n. with ORF73^−^Cre^−^ MuHV-4, then 2 months later challenged i.p. with Cre^+^ MuHV-4 as in (a). The data are from one of two equivalent experiments. (c) In an equivalent experiment to (b), spleens were were analysed for viral DNA content by quantitative PCR 1 month after Cre^+^ virus challenge. Viral genomes per cell genome are shown for each mouse (means of three replicate reactions). The dashed line shows the sensitivity limit of one viral genome per 500 cell genomes.

The high efficiency of virus-triggered oncogenesis in our model suggested that vaccine-induced protection might be difficult to achieve. However, when Cre was substituted for ORF50 to make a replication-deficient Cre^+^ MuHV-4, both i.p. and intranasal (i.n.) infections gave no disease in p53^flox/flox^K-ras^LSL-G12D/+^ mice over 5 months (*n*=30). This lack of disease without lytic spread suggested that vaccination might still work – for example, the cells first encountered by incoming virions might not be transformed by k-ras. We therefore immunized p53^flox/flox^K-ras^LSL-G12D/+^ mice either i.n. or i.p. with ORF73^−^Cre^−^ MuHV-4, which lacks episome maintenance and so fails to persist *in vivo* (Fowler *et al.*, 2003; Moorman *et al.*, 2003). This protected completely against Cre^+^ virus challenge (Fig. 6).

**Fig. 7.**
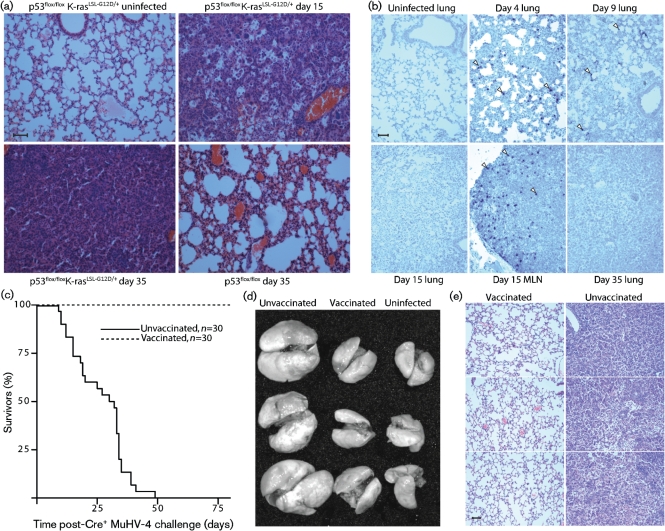
Vaccination against i.n. Cre^+^ MuHV-4 challenge. (a) p53^flox/flox^K-ras^LSL-G12D/+^ or p53^flox/flox^ mice were infected i.n. with Cre^+^ MuHV-4. Lungs were examined by haematoxylin/eosin staining at 15 or 35 days post-infection. The p53^flox/flox^ mice showed moderate abnormalities but remained clinically well. Bar, 100 μm. Sections are representative of at least six mice per group. (b) p53^flox/flox^K-ras^LSL-G12D/+^ mice were not infected or infected i.n. with Cre^+^ MuHV-4. Lungs and mediastinal lymph nodes (MLN) were analysed for viral tRNAs by *in situ* hybridization. The sections are each representative of at least five mice per group. The arrows show examples of positive cells. Bar, 100 μm. (c) p53^flox/flox^K-ras^LSL-G12D/+^ mice were vaccinated i.p. with ORF73^−^Cre^−^ MuHV-4, and 2 months later challenged i.n. with Cre^+^ MuHV-4. Mice were killed when they showed >20 % weight loss or progressive respiratory difficulties. The vaccinated mice remained entirely well. Equivalent data were obtained in one further experiment. (d) *Ex vivo* p53^flox/flox^K-ras^LSL-G12D/+^ lungs (three per group) are shown 1 month after i.n. Cre^+^ MuHV-4, after the same challenge but vaccinated i.p. with Cre^−^ORF73^−^ MuHV-4 2 months earlier, or without infection. Equivalent results were obtained in three further experiments. (e) Lungs of p53^flox/flox^K-ras^LSL-G12D/+^ mice were examined by haematoxylin/eosin staining 35 days post-infection with Cre^+^ MuHV-4. The lungs of vaccinated mice were macroscopically and histologically normal. Three representative images are shown for each group. Equivalent results were obtained in two further experiments, each with five mice per group. Bar, 100 μm.

**Fig. 8.**
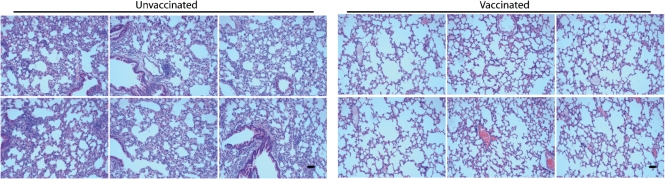
Protection of p53^flox/flox^ mice against i.n. Cre^+^ MuHV-4 by an ORF73^−^Cre^−^ vaccine. p53^flox/flox^ mice were not vaccinated or vaccinated i.p. with ORF73^−^Cre^−^ MuHV-4, then 3 months later challenged i.n. with ORF73^+^Cre^+^ MuHV-4. Lungs were examined histologically at 1 month post-challenge. Equivalent p53^flox/flox^K-ras^LSL-G12D/+^ lungs are shown in Fig. 7. Bars, 100 μm. The results are representative of >15 mice per group from three independent experiments.

As a further test of vaccine efficacy, we established an i.n. Cre^+^ virus challenge model (Fig. 7). This caused a more rapid illness than i.p. infection, with weight loss and respiratory difficulties as early as 7 days post-inoculation. The lungs of infected mice became grossly enlarged, and histological examination (Fig. 7a) showed extensive cell proliferation obliterating the alveolar air spaces. p53^flox/flox^K-ras^LSL-G12D/+^ mice infected with Cre^−^ MuHV-4 and p53^flox/flox^ mice infected with Cre^+^ MuHV-4 remained clinically well, so disease again reflected k-ras activation. *In situ* hybridization (Fig. 7b) showed viral tRNA expression in acutely infected lungs and lymphoid tissue, but not in diseased lungs. Therefore, viral genomes were again lost rapidly from the transformed cells. Vaccination i.p. with Cre^−^ORF73^−^ MuHV-4 protected completely against both macroscopic and microscopic disease (Fig. 7c–e). It also protected against the milder histological changes induced by Cre^+^ MuHV-4 in p53^flox/flox^ mice (Fig. 8).

## DISCUSSION

A viral aetiology is rarely considered for cancers that lack viral genomes. Our data show that cells driven to proliferate by host oncogenes readily lose gammaherpesvirus genomes *in vivo*. Relying on viral genome detection to establish aetiology could therefore underestimate the number of cancers to which gammaherpesviruses contribute. Most analyses of human cancers have focused on examples of genome retention. The hypothesis that these viral genomes contribute to disease (Hammerschmidt & Sugden, 2004) makes sense, as there must be a growth advantage to offset any immune recognition of viral antigens. Thus, whilst EBV genes seem not to drive the growth of EBV^+^ Burkitt's lymphoma directly (Kang *et al.*, 2005), they may still provide important co-factors (Thorley-Lawson & Allday, 2008). However, the retention of viral genomes by some cancer types does not establish that viral genome retention is the norm. Interestingly, whilst EBV^+^ Burkitt's lymphoma is associated strongly with immunosuppressive malaria infection, EBV^−^ Burkitt's lymphoma occurs later and shows no such association. Thus, in immunocompetent hosts, EBV genome loss may be required for cancers to evolve.

Viral antigen recognition (Rickinson & Moss, 1997) provides a context for understanding both genome-positive and genome-negative cancers. Cells driven to proliferate by the EBV growth programme are normally killed by antiviral T cells, so EBV-driven cancers are limited to the immunocompromised. In contrast, host mutations drive non-immunogenic cell proliferation even when the viral growth programme is turned off. This creates a new balance: viral genes are now required only for accessory roles, allowing viral antigen recognition to be reduced. However, some immune control may still occur – for example, the evasion of antigen presentation by gammaherpesvirus episome-maintenance proteins (Yin *et al.*, 2003; Bennett *et al.*, 2005) can fail at high proliferation rates (Münz, 2004). Also, the accumulation of host mutations is unlikely to stop. If host mutations alone remain insufficient to maintain transformation, cancer cells losing viral genomes will themselves be lost; however, if host mutations become sufficient, then antiviral T cells can select for viral genome loss.

The predominance of sarcomas in our model was surprising, as MuHV-4 classically persists in B cells (Sunil-Chandra *et al.*, 1992). However, stromal cells may also be an important site of persistence (Stewart *et al.*, 1998; Suárez and van Dyk, 2008) – consistent with such an idea, ORF50^−^ MuHV-4 genomes were well-maintained over 3 weeks in both BHK-21 and p53^−/−^K-ras^LSL-G12D/+^ fibroblasts (data not shown). Stromal cells may also be more sensitive than B cells to transformation by k-ras (Nicolaides *et al.*, 1994; Janssen *et al.*, 2005). A key point is that known viral tropisms do not necessarily predict the cell type of virus-triggered cancers. Thus, hit-and-run oncogenesis may be more relevant to rarely EBV^+^ cancers such as gastric adenocarcinoma (Deyrup, 2008; Shah & Young, 2009) than to those of B cells. Even in transformed fibroblasts, MuHV-4 (unlike HSV) is far from uniformly lytic (May *et al.*, 2004), and productive MuHV-4 spread is strongly constrained *in vivo* by host immunity. Therefore, it would seem quite feasible for a virus-positive cancer to develop in a cell type permissive for lytic replication.

There is no certain way to identify a human cancer as previously virus-positive once it becomes virus-negative, so human gammaherpesvirus disease burdens may only be revealed by vaccination. This is not necessarily straightforward: subunit vaccines have so far failed to limit gammaherpesvirus persistence (Sokal *et al.*, 2007; Stevenson *et al.*, 2009). However, live-attenuated vaccines can reduce MuHV-4 latent loads (Tibbetts *et al.*, 2003; Boname *et al.*, 2004; Fowler & Efstathiou, 2004; Rickabaugh *et al.*, 2004). Here, we extended this protection to a high-penetrance cancer. Latency-deficient EBV and KSHV vaccines therefore deserve serious consideration. The possibility that gammaherpesviruses contribute to more cancers than simply those remaining viral genome-positive argues that such vaccines might greatly benefit human health.

## METHODS

### Mice.

p53^flox/flox^ (Marino *et al.*, 2000), K-ras^LSL-G12D/+^ (Jackson *et al.*, 2001) and ROSA26-*lacZ*^flox/flox^ (Soriano, 1999) mice were infected with MuHV-4 either i.n. under general anaesthesia (10^4^ p.f.u.) or i.p. (10^6^ p.f.u.). All experiments conformed to local and national ethical regulations. Mice were killed when they showed macroscopic cancers or other signs of ill health. All mice were examined post-mortem for clinically inapparent cancers. The PCR primer sequences for detecting *loxP* recombination were: p53 – 5′-CACAAAAACAGGTTAAACCCAG and 5′-GAAGACAGAAAAGGGGAGGG to detect only the recombined locus (612 bp); and k-ras – 5′-CCATGGCTTGAGTAAGTCTGC and 5′-CGCAGACTGTAGAGCAGCG to detect the ‘floxed’ (flanked by *loxP* sites) G12D k-ras cassette (550 bp) before but not after recombination, or 5′-GTCTTTCCCCAGCACAGTGC, 5′-CTCTTGCCTACGCCACCAGCTC and 5′-AGCTAGCCACCATGGCTTGAGTAAGTCTGCA to amplify from the floxed G12D k-ras cassette a 500 bp band before recombination and a 650 bp band after recombination.

### Cells.

For *ex vivo* explants, tissues were minced finely and digested with trypsin before culture. Embryonic fibroblasts were derived from 14 day embryos. All cells were grown in Dulbecco's modified Eagle's medium supplemented with 10 % fetal calf serum, 2 mM glutamine, 50 μM *β*-mercaptoethanol (Sigma), 100 U penicillin ml^−1^ and 100 μg streptomycin ml^−1^. All media and reagents listed here except *β*-mercaptoethanol were from PAA Laboratories GmbH.

### Viruses.

ORF73^−^ MuHV-4 has been described previously (Fowler *et al.*, 2003). To make Cre^+^ MuHV-4, an HCMV IE1 promoter-driven Cre expression cassette was excised from pGS403 (Smith & Enquist, 2000) with *Sal*I/*Sac*II, end-repaired and cloned into the intergenic *Mfe*I site (genomic co-ordinate 77176 of GenBank accession no. U97553) of a *Bgl*II MuHV-4 genomic clone (co-ordinates 75338–78717). All other genomic co-ordinates are also given relative to GenBank accession no. U97553. The Cre expression cassette plus genomic flanks was then subcloned with *Sph*I/*Sca*I (78413–75785) into the *Sph*I/*Sma*I sites of pST76K-SR and recombined into an MuHV-4 BAC (Adler *et al.*, 2000). Infectious virus was recovered by transfecting BAC DNA into BHK-21 cells. The BAC cassette was removed by virus passage through NIH-3T3-CRE cells (Stevenson *et al.*, 2002) and virus stocks were grown in BHK-21 cells (de Lima *et al.*, 2004). Replication-deficient, Cre^+^ MuHV-4 was made by digesting a *Hin*cII genomic fragment (63844–70433) in pUC9 with *Bsm*I (67792) and *Cla*I (69177) to remove most of ORF50 exon 2 (67661–69376). The Cre coding sequence plus a 3′ poly(A) site from pGS403 was ligated in its place in frame with the ORF50 AUG. The Cre coding sequence plus genomic flanks (66120–70433) was then subcloned with *Kpn*I into pST76K-SR, and recombined into the MuHV-4 BAC. ORF50^−^Cre^+^ virus was recovered by transfecting BAC DNA into NIH-3T3-TET50 cells and inducing ORF50 expression with doxycycline (Milho *et al.*, 2009).

### Virus assays.

Virus stocks were titrated by plaque assay on BHK-21 cells (de Lima *et al.*, 2004). Latent virus was measured by infectious centre assay (de Lima *et al.*, 2004). Plaque titres of freeze–thawed spleen cells were always <1 % of infectious centre assay titres. Viral genome loads were measured by quantitative PCR (Milho *et al.*, 2009). Briefly, MuHV-4 genomic co-ordinates 4166–4252 were amplified from 50–100 ng DNA and quantified by hybridization with a Taqman probe (genomic coordinates 4218–4189) (Rotor Gene 3000; Corbett Research), in comparison with a standard curve of cloned plasmid template amplified in parallel. Cellular DNA was quantified in the same way by amplifying part of the adenosine phosphoribosyltransferase gene (forward primer, 5′-GGGGCAAAACCAAAAAAGGA; reverse primer, 5′-TGTGTGTGGGGCCTGAGTC; probe, 5′-TGCCTAAACACAAGCATCCCTACCTCAA).

To quantify viral DNA by Southern blotting, DNA was extracted from cells (Wizard Genomic DNA purification kit; Promega), digested with *Pst*I, electrophoresed, transferred to Hybond nylon membranes (Roche Diagnostics), then probed with a [^32^P]dCTP random-primed 1.2 kb *Pst*I genomic fragment corresponding to the MuHV-4 terminal repeat unit (Efstathiou *et al.*, 1990), washed (65 °C, 0.2 % SSC, 0.1 % SDS) and exposed to X-ray film. Recombinant viruses were analysed qualitatively for genomic structure in a similar way, except that viral DNA was digested with *Bgl*II or *Hin*dIII and probed with a *Bgl*II-restricted genomic fragment (co-ordinates 75338–78717) or the HCMV IE1–Cre construct.

Cells expressing viral tRNAs 1–4 were detected by *in situ* hybridization of formaldehyde-fixed, paraffin-embedded spleen cell sections, using a digoxigenin-labelled riboprobe transcribed from pEH1.4 (Bowden *et al.*, 1997). Hybridized probe was detected with alkaline phosphatase-conjugated anti-digoxigenin Fab fragments (Roche Diagnostics).

### *β*-Galactosidase assay.

*In vitro* samples were fixed in 4 % formaldehyde (30 min), then washed in PBS and incubated (3 h, 37 °C) in PBS with 0.01 % sodium deoxycholate, 0.02 % Nonidet P-40, 2 mM MgCl_2_, 4.5 mM potassium ferricyanide, 4.5 mM potassium ferrocyanide, 1 mg X-Gal ml^−1^, before washing. *In vivo* samples were fixed in 4 % formaldehyde (18 h) then frozen in OCT medium, sectioned, washed in PBS and developed as described above before washing and mounting.

### Immunofluorescence.

Cells were plated onto glass cover slides, then fixed (4 % formaldehyde, 30 min), permeabilized (0.1 % Triton X-100, 15 min), blocked (3 % BSA in PBS, 15 min) and stained for syndecan-1, CD44, VCAM-1 (all mAbs from BD Biosciences) or with the macrophage-specific mAb F4/80 (AbCam) plus Alexa Fluor 568-conjugated goat anti-rat IgG pAb (Invitrogen), for the MuHV-4 ORF75c using mAb BN-6C12 (Gaspar *et al.*, 2008) plus Alexa Fluor 568-conjugated goat anti-mouse IgG pAb (Invitrogen), for MuHV-4 antigens using a polyclonal rabbit serum (Sunil-Chandra *et al.*, 1992) and for Cre recombinase using a polyclonal rabbit serum (AbCam) plus goat anti-rabbit IgG pAb (Invitrogen). The cells were mounted in ProLong Gold anti-fade reagent with DAPI (Invitrogen) and imaged using an Olympus IX70 microscope plus a Retiga 2000R camera line (QImaging).
